# Mesocorticolimbic Interactions Mediate fMRI-Guided Regulation of Self-Generated Affective States

**DOI:** 10.3390/brainsci10040223

**Published:** 2020-04-08

**Authors:** Andrea Caria

**Affiliations:** Department of Psychology and Cognitive Sciences, University of Trento, Corso Bettini 33, 38068 Rovereto, Italy; andrea.caria@unitn.it; Tel.: +39-0464808465; Fax: +39-0464808402

**Keywords:** emotion regulation, midbrain, anterior insula, periaqueductal gray, reinforcement learning, real-time fMRI

## Abstract

Increasing evidence shows that the generation and regulation of affective responses is associated with activity of large brain networks that also include phylogenetically older regions in the brainstem. Mesencephalic regions not only control autonomic responses but also participate in the modulation of autonomic, emotional, and motivational responses. The specific contribution of the midbrain to emotion regulation in humans remains elusive. Neuroimaging studies grounding on appraisal models of emotion emphasize a major role of prefrontal cortex in modulating emotion-related cortical and subcortical regions but usually neglect the contribution of the midbrain and other brainstem regions. Here, the role of mesolimbic and mesocortical networks in core affect generation and regulation was explored during emotion regulation guided by real-time fMRI feedback of the anterior insula activity. The fMRI and functional connectivity analysis revealed that the upper midbrain significantly contributes to emotion regulation in humans. Moreover, differential functional interactions between the dopaminergic mesocorticolimbic system and frontoparietal networks mediate up and down emotion regulatory processes. Finally, these findings further indicate the potential of real-time fMRI feedback approach in guiding core affect regulation.

## 1. Introduction

Emotion generation and regulation shape our perception, cognition, and behavior. Emotion regulation refers to a variety of regulatory processes, either explicit or implicit, involving the physiological, expressive, and experiential components of emotion that permit the selection and implementation of appropriate responses to the environment. Adaptive emotion regulation strategies characterize healthy behavior whereas impairment in experiencing and regulating emotion often leads to psychopathological disorders [1]. Increasing evidence shows that regulation of affective responses is associated not only with the activity of cortical brain networks [2,3] but also involves phylogenetically older regions in the brainstem [4,5,6]. A considerable amount of data from brain lesions, neural recordings, and neuroimaging studies demonstrates that mesencephalic regions and other brainstem regions participate in emotion generation and regulation [7,8,9]. For instance, the ventral tegmental area (VTA)/substantia nigra (SNG) complex exerts an important modulatory function of autonomic, emotional, and motivational responses through ascending dopaminergic nigrostriatal and mesocorticolimbic pathways [10,11]. In addition, the periaqueductal grey (PAG), a mesencephalic region strongly involved in fear responses [12], would also mediate emotional behavior through stimulus salience processing [5,13].

To date, the specific contribution of brainstem areas to emotion generation and regulation in humans remains elusive and not sufficiently investigated. However, it has been shown that a first level of integrated representation of sensory, motor, and modulatory signals related to emotional behavior occurs at this level [8,14]. Further integration of body signals is then vertically operated in subcortical and cortical level systems such as the thalamus and the insular cortex. Notably, ascending pathways from the brainstem to the anterior insula (AI) have been proposed to mediate socio-affective behavior [15] and interoception [16]. In fact, the midbrain and anterior insula, two important nodes of the so-called salience network (SN) [17], regulate homeostasis and core affect generation [2,6]. Specifically, the SN, also including the dorsomedial prefrontal cortex, the ventral striatum, and the VTA/SNG complex, primarily contributes to both exogenous and endogenous emotion generation [2,6]. 

Neuroimaging studies grounding on appraisal models of emotion emphasize a major role of forebrain regions, such as the lateral and medial prefrontal cortex and the anterior cingulate cortex, for cognitive emotion regulation, but the contribution of other regions such as the parietal cortex, insula, amygdala, and striatum is also observed [18,19,20,21,22]. The midbrain and other brainstem regions despite their clear involvement in emotion generation are usually not included in the emotion regulation network. In addition, reinforcement learning accounts of emotion regulation [23] also postulate that the midbrain would support regulatory processes by mediating dopaminergic prediction error signals resulting from the mismatch between expected and actual emotional state [24,25]. Nevertheless, clear evidences on the role of upper mesencephalic regions in the regulation of affective states are still lacking.

In addition to the cognitive approach to emotion regulation, an alternative method consists in regulating emotion guided by online feedback of the BOLD signal magnitude in relevant brain circuits [26,27]. Several real-time fMRI investigations demonstrated that healthy individuals and patients can attain specific control of cortical and subcortical areas involved in emotional processing [28,29,30,31,32,33,34,35,36,37,38,39,40,41], including dopaminergic midbrain regions such as the VTA and SNG [42,43,44]. 

Here, I aimed to unveil the role of human midbrain, as well as of mesolimbic and mesocortical networks interactions in supporting core affect generation and regulation. To this purpose, data from two previous emotion regulation studies, where participants successfully regulated the AI activity with a combination of emotional strategies and real-time fMRI technique, were considered [28,41]. In both studies participants were instructed to endogenously generate emotions by recalling and reactivating subjective physiological and experiential components of affective memory [6,15,45], and to modulate them on the basis of fMRI feedback information. Real-time fMRI feedback, covertly to the participants, represented the BOLD signal amplitude of the AI, an important node of the SN that mediates a subjective evaluation of emotional intensity [17,46,47]. This fMRI-guided regulatory process by combining top-down mental imagery strategies with bottom-up information of AI activation was expected to rely on core mechanisms of affect regulation that are here assumed to engage the midbrain activity.

## 2. Methods

Seventeen right-handed participants from two previous studies [28,41] (nine subjects from Caria et al. 2007 and eight subjects from Caria et al. 2010; nine women; mean age = 25.13, SD = 4.09) were included based on the MR field of view coverage encompassing the upper portion of the brainstem, and on the adoption of regulation strategies focusing on recalling of emotional memories. All participants had no history of neurological or psychiatric disorders including substance abuse/dependence and psychotropic medications. Both studies were approved by the local ethics committee of the University of Tübingen.

### 2.1. fMRI Data Acquisition

All functional images were acquired using a 3.0 T MR Siemens scanner, with a 12 channels head coil (Siemens Magnetom Trio Tim, Siemens, Erlangen, Germany. During real-time fMRI-based emotion regulation, standard echo planar imaging (EPI) images consisting of sixteen axially oriented slices (voxel size = 3 × 3 × 5 mm^3^, slice gap = 1 mm) were acquired (repetition time TR = 1500 ms, matrix size = 64 × 64, FoV = 192 × 192 mm, TE = 30 ms). Considering that the primary respiratory-related component of the fMRI signal usually fluctuates at about 0.3 Hz, a TR equal to 1.5 s prevents aliasing of the first respiratory harmonics with a spectral signature of the typical BOLD effect [48]. Additionally, a gradient echo field map (TR 488 ms, TE 1 = 4.49 ms, TE 2 = 6.95 ms) and a T1-weighted MPRAGE structural scan (matrix size = 256 × 256, 160 partitions, 1 mm^3^ isotropic voxels, TR = 2300 ms, TE = 3.93 ms, TI = 1100 ms, α = 8°) were acquired from each participant to reduce geometric distortion due to magnetic field inhomogeneities [49]. In order to minimize head movements, two foam cushions were positioned around the participant’s head.

### 2.2. Real-Time fMRI Analysis 

MR images were exported online from the MR console computer to a separate computer for real-time preprocessing and analysis with the Turbo brain voyager (Brain Innovation, Maastricht, The Netherlands). Online preprocessing included incremental 3D motion correction and drift correction. Incremental statistical data analysis was based on the recursive least squares general linear model (GLM). The selection of the target regions-of-interest (right AI, n = 9, from Caria et al. 2007 and left AI, n = 8, from Caria et al. 2010) was performed anatomically based on the high resolution T1 structural scan for the right AI and consisted of a rectangular area of 4 × 5 voxels on a single slice; anatomically and functionally, through a localizer session consisting of five alternating emotional recall and baseline blocks, for the left AI and consisted of a rectangular area of 5 × 5 voxels on a single slice. A similar reference region -of-interest consisting of a large background area not encompassing emotion-related areas and used to cancel out global effects and unspecific activations was considered in both studies. During training, the averaged BOLD signal in the AI normalized with respect to the reference region [28,41] was calculated during *up emotion regulation* with respect to *down emotion regulation*, considering three consecutive TRs in order to reduce rapid signal fluctuations, and excluding the first ten volumes of each session to account for T1 equilibration effects. Participants were provided with online continuous fMRI information of the AI activity through a visual feedback consisting of a graduated thermometer displaying changes of BOLD response with increasing or decreasing number of bars updated every 1.5 s.

### 2.3. Experimental Protocol

The real-time fMRI-based emotion regulation training consisted of emotion regulation blocks divided in four runs (16 blocks for n = 9; 20 blocks for n = 8) performed in one day. Each run consisted of *up emotion regulation* blocks (22.5 s n = 9; 30 s n = 8), cued with an arrow at the right side of the thermometer, alternating with *down emotion regulation* blocks (22.5 s n = 9; 30 s n = 8), cued with a cross hair at the right side of the thermometer. In both studies, participants were instructed that during *up- and down- regulation* blocks they had to modulate the intensity of recalled emotional memories and imagery of personally relevant affective episodes guided by increasing or decreasing the number of thermometer bars, that represented the level of brain activation in an unspecified region. Participants were informed that the feedback information, provided in the form of thermometer bars, was delayed for about 1.5 s due to online data analysis and physiological latency of the hemodynamic response. Participants were requested not to move during all the experimental conditions, and informed that physiological signals were monitored.

### 2.4. fMRI Analysis

The fMRI data preprocessing and analysis were performed using SPM12 (Wellcome Trust Centre for Neuroimaging, London, UK) and MATLAB (The MathWorks, Inc., Natick, MA, US). For each participant, all functional images were first realigned to the mean image using least squares and a six parameter (translations and rotations in space) and including resampling using the second degree B-spline interpolation, then unwarped and corrected for geometric distortions using the fieldmap of each participant. The high-resolution T1 image was coregistered to the mean image of the EPI series using a rigid body model, estimated with mutual information. Segmentation parameters were used to normalize the functional images to the Montreal Neurological Institute (MNI) space. Lastly, normalized images were spatially smoothed with a 6 mm FWHM Gaussian kernel in order to balance effect size, spatial accuracy, and statistical significance estimated using Gaussian random fields [50,51]. 

A fixed-effects general linear model (GLM) was used to perform the first-level statistical analysis. Hemodynamic response amplitudes were estimated using standard regressors, constructed by convolving a boxcar function, for up and down emotion regulation, with a canonical hemodynamic response function using standard SPM12 parameters. The time series in each voxel were high-pass filtered at 1/128 s to remove low frequency drifts. An autoregressive AR (1) model was employed to address autocorrelation in the time series. Contrast images of up emotion regulation versus down emotion regulation were created for each block and run. Movement parameters were also included in the GLM as covariates to account for head motion artifacts. The second-level random effects group analysis was performed by entering single subject contrast images into one sample *t*-tests. Whole brain statistical maps were thresholded at *p* < 0.001 corrected at the cluster level for multiple comparison using a probabilistic threshold-free cluster enhancement (pTFCE) [52,53], an approach that integrates cluster information into voxel-wise statistical inference so as to enhance detectability of the neuroimaging signal and to control for the Type I error. In order to increase the statistical power of fMRI analysis, considering the relatively limited sample size, hypothesis-driven regions-of-interest (ROIs) statistical analysis was first performed [54,55]. Three independent ROIs were delineated, using the MarsBar toolbox (http://marsbar.sourceforge.net) [56,57], in the bilateral VTA (two 6 mm radius spheres centered at ±4, −14, −10), in the bilateral SNG (two 6 mm radius spheres centered at ±8, −18, −4), and in the PGA (9 mm radius sphere centered at ±2, −30, −10). ROIs were defined based on cluster peaks of uniformity maps generated from an automated meta-analysis of studies (75 studies for VTA, 74 for SNG, and 80 for PGA) using Neurosynth (https://www.neurosynth.org), search terms were ventral tegmental, substantia, and periaqueductal, respectively). Involvement of ROIs during fMRI-guided emotion regulation was tested by assessing a single subject’s average β-values for the contrast up > down emotion regulation using the bootstrap analysis (1000 bootstrap samples, 95% bias corrected and accelerated confidence interval), as implemented in the SPSS statistics software (v. 24, IBM Corp. Armonk, NY, USA). In addition, the whole brain activity was also considered to assess the specificity of midbrain activation, that was estimated by calculating a selectivity index [55], that is the proportion of all voxels showing increased activity during emotion regulation. Finally, engagement of the frontoparietal control network (FPCN), known to play a main role in the cognitive control of emotion [18,19,20,21,22], was explored using a less stringent threshold (*p* < 0.005 pTFCE corrected). 

### 2.5. fMRI Connectivity Analysis

The spatiotemporal dynamic behavior of functional connectivity patterns involving the upper midbrain were explored during a short emotion regulation timescale. Although largely debated, the task-based modulation of functional connectivity has been shown to be cognitively meaningful and not artefactual [58,59]. In fact, previous investigations demonstrated a robust association between functional connectivity states and ongoing cognition, permitting for instance accurate detection of mental states over a time window of tens of seconds [60,61]. 

The functional connectivity analysis was then conducted by integrating multivariate and univariate approaches so as to enhance results reliability. First, the group-wise independent component analysis (group-ICA) aimed to detect the engagement of the midbrain region during real-time fMRI-guided emotion regulation. Second, seed-to-voxel connectivity maps assessed specific whole brain functional interactions of the midbrain. In addition, whole brain functional connectivity of the right AI, a region considered a dynamic hub for salient information processing [47], was also estimated. 

Multivariate and univariate connectivity analyses were performed using the CONN functional connectivity toolbox (Release 18.a, http://www.nitrc.org/projects/conn) [62,63]. Before computing the connectivity analysis, denoising was applied to reduce motion, physiological, and additional artefactual effect from the BOLD signal. Noise reduction was performed based on the available CompCor method that includes the principal components (five each) of WM and CSF time series as nuisance covariates; WM and CSF were identified via segmentation of the anatomical images with SPM12. The six head motion parameters derived from spatial motion correction were also added as confounds. Band-pass filtering with a frequency window of 0.008 to 0.09 Hz was applied.

### 2.6. Independent Component Analysis

Preprocessed images were submitted to a subject-wise group-ICA to identify networks of highly functionally-connected areas. The CONN toolbox implementation uses Calhoun’s group-level ICA approach [64] with variance normalization preconditioning, subject-level dimensionality reduction, subject/condition concatenation of BOLD signal data along temporal dimension, group-level dimensionality reduction (to the target number of dimensions/components), fastICA for estimation of independent spatial components, and GICA1 back-projection for individual subject-level spatial map estimation. The number of independent components estimated was 20 and the degree of subject-level dimensionality reduction (number of subject-specific SVD components retained when characterizing the voxel-to-voxel connectivity matrix for each subject/condition was 64. Reliability of the resulting independent components was based on a conservative group level statistical threshold set to *p* < 0.001 voxel level FDR corrected.

### 2.7. Seed-to-Voxel Connectivity 

A weighted GLM for weighted temporal correlation measures of the real-time fMRI-based emotion regulation-specific association between a *seed* BOLD time series and each voxel BOLD time series was considered. Seed-to-voxel connectivity maps were created for each participant. The *seeds* consisted of the activity within a 9 mm spherical region in the right upper *midbrain* (centered at 2, −30, −10) defined using Neurosynth (see the previous fMRI analysis section), and a predefined right AI node of the SN (47, 14, 0; available on the CONN toolbox), masked with an unthresholded subject-specific estimated gray matter mask. Bivariate-correlation analyses were used to determine the linear association of the BOLD time series between the two defined ROIs, separately and whole brain areas on each participant. Individual seed-to-voxel maps were then entered into a second-level analysis. Temporal correlations were analyzed separately for *up* and *down emotion regulation* runs. Significant correlations were assessed considering a threshold of *p* < 0.001 cluster level corrected for multiple comparison using pTFCE [52,53].

## 3. Results

### 3.1. ROI Analysis

**Figure 1:**
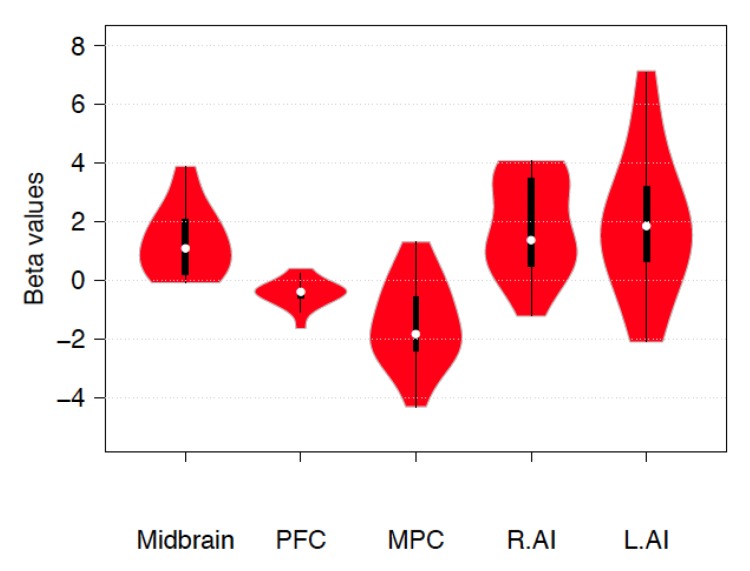
Violin plots representing estimated beta values for the contrast *up > down emotion regulation* in the midbrain (5, −26, −10), prefrontal cortex (PFC, −21, 30, −10), medial parietal cortex (MPC, 2, −30, 50), and left and right AI (−37, 20, 0 and 42, 13, 5). White circles show the medians; box limits indicate the 25th and 75th percentiles as determined by the R software; whiskers extend 1.5 times the interquartile range from the 25th and 75th percentiles; polygons represent density estimates of beta values and extend to extreme values. Violin plots were created with BoxPLotR (http://shiny.chemgrid.org/boxplotr/) [65].

A significant increase during emotion regulation was observed in the PAG (β = 1.323 ±1.53SD, *p* = 0.003, confidence interval 0.65–2.05) and in the SNG (β = 0.813 ± 1.53 SD, *p* = 0.05, confidence interval 0.16–1.56) but not in the VTA (β = 0.673 ± 1.83 SD, *p* = 0.168). Additional β-values for the contrast up > down emotion regulation in the midbrain, left and right AI, and FPCN are reported in Figure 1. 

### 3.2. Whole Brain Group Analysis

**Figure 2:**
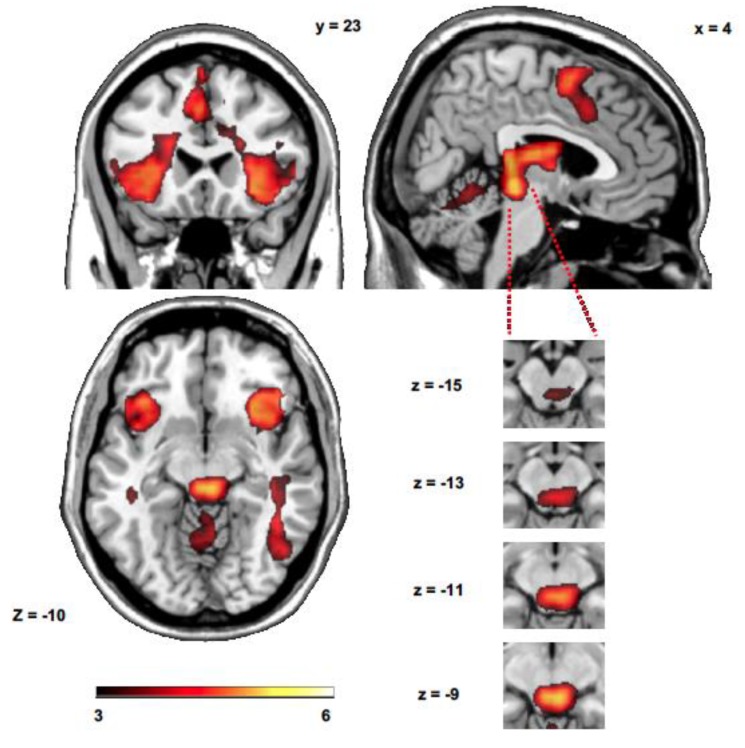
SPM maps showing the whole brain and specific midbrain activity during *up emotion regulation* with respect to *down emotion regulation*. Activation maps are superimposed on a Ch2 template using the MRIcron software (version 2016) [66].

The sensitivity index calculation revealed that 10.95% of all voxels showed a significant increase during *up* > *down emotion regulation* (*p* < 0.001 cluster level corrected using pTFCE). The analysis of all *up* > *down emotion regulation* runs showed brain activity in the left and right AI, in the right middle temporal gyrus, premotor cortex and SMA, midbrain (PAG/superior colliculus), caudate nucleus and thalamus (Figure 2 and Table 1). The analysis of *down > up emotion regulation* runs did not show significant differences at *p* < 0.001 pTFCE corrected. However, additional activated clusters in the prefrontal cortex (−21, 30, −10, *t* = 3.06) and in the parietal cortex (2, −29, 50, *t* = 3.00) were also observed at *p* < 0.005 pTFCE corrected.

### 3.3. Functional Connectivity—ICA

**Figure 3:**
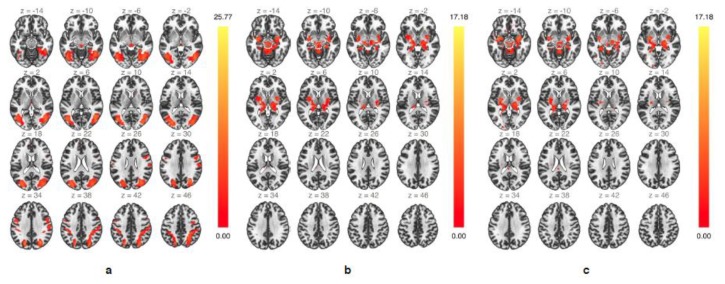
Group-independent component analysis (ICA) showing highly functionally-connected networks involving mesencephalic areas during *up emotion regulation* (**a**,**b**) and during *down emotion regulation* (**c**).

The group-ICA analysis during *up emotion regulation* revealed two specific networks of highly functionally-connected clusters involving mesencephalic areas (Figure 3). A first bilateral network consisted of mainly subcortical areas within the SN and included the upper midbrain, insular cortex, amygdala, hippocampus and parahippocampal gyrus, thalamus, and putamen (Figure 3a). A second bilateral network consisting of several occipital, parietal, and temporal regions, many of them associated with semantic and episodic memory retrieval [67,68,69], included the lateral occipital cortex, fusiform area, superior parietal lobule, supramarginal gyrus, superior, middle and inferior temporal gyrus, precuneus, and upper midbrain (Figure 3b). During *down emotion regulation* only one single network involved the midbrain, and similarly to that observed during *up emotion regulation* included subcortical areas of the salience network (Figure 3c). Taken together, the group-ICA identified several functionally interconnected core areas of the extended salience network during real-time fMRI-guided emotion regulation.

### 3.4. Functional Connectivity—Seed-To-Voxel

**Figure 4:**
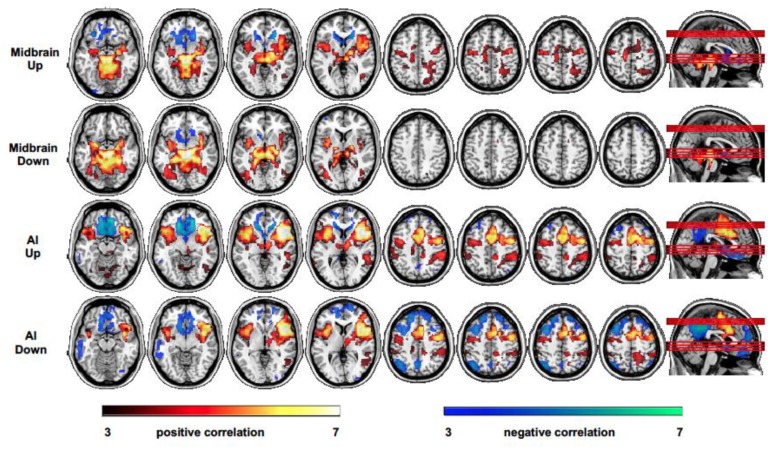
Patterns of functional connectivity measured as a positive and negative correlation of the midbrain with the whole brain (seed-to-voxel connectivity maps) during *up and down emotion regulation,* separately (first and second row); positive and negative correlation of the AI with the whole brain during *up and down emotion regulation,* separately (third and fourth row)**.**

During *up emotion regulation,* the midbrain showed a positive correlation mainly with the left and right anterior insular cortex and a bilateral pattern of subcortical regions including other upper mesencephalic regions, such as the VTA, SNG, thalamus and striatum, hippocampus and parahippocampal gyri, amygdalae, in addition to the left superior and middle temporal gyrus, posterior cingulate, premotor regions, SMA, and cerebellum (Figure 4, first row); a negative correlation was observed with the inferior orbitofrontal cortex (BA11), medial prefrontal cortex (BA10), bilateral caudate and nucleus accumbens, and subgenual anterior cingulate cortex (Figure 4, first row). The right AI showed a positive correlation with several regions including left AI, anterior and middle cingulate, the right thalamus, left and right putamen, the right midbrain (PAG), bilateral superior temporal gyrus, the premotor cortex and SMA, the right supramarginal gyrus, and right middle frontal gyrus (BA10) (Figure 4, second row); a negative correlation was observed with the medial orbitofrontal cortex (BA11), medial prefrontal cortex (BA10), anterior cingulate, right angular gyrus, posterior cingulate, and precuneus (Figure 4, second row). 

During *down emotion regulation,* the midbrain showed a pattern of correlation and anticorrelation similar to that observed during up emotion regulation with reduced correlation (but not significant, up vs. down comparison) with the bilateral AI *(*Figure 4, third row). The right AI also showed a pattern of correlation and anticorrelation similar to that observed during *up emotion regulation,* but no coupling with the midbrain was observed (Figure 4, fourth row). A significantly larger positive correlation with the posterior cingulate cortex and dorsomedial prefrontal cortex was measured during up with respect to down emotion regulation. 

## 4. Discussion

The current study explored the role of the midbrain and its functional interactions within mesolimbic and mesocortical brain systems during emotion regulation. To this aim, the region of interest fMRI analysis and functional brain connectivity estimation was performed during AI-mediated self-regulation of endogenously-generated emotion. The ROI-based and whole brain analysis showed that emotion regulation guided by feedback of AI activity is mediated by the midbrain. Specifically, up regulation of emotion as compared to down regulation engaged important nodes of the SN mediating emotional generation and homeostasis such as the upper midbrain and AI [2,15]. Down regulation of affective response with respect to up regulation was instead mainly associated with activity in the medial prefrontal cortex, posterior cingulate, and parietal cortex regions known to be engaged during retrieval of neutral autobiographical memories and self-referential thinking [70,71,72], mental processes indeed adopted by the participants during this condition. 

No upper mesencephalic activity is typically reported in cognitive emotion regulation studies, but there exist some previous evidences indicating midbrain involvement during real-time fMRI-based emotion regulation training in healthy individuals and patients [73,74,75,76]. For instance, in healthy individuals, up regulation of the right AI mediated by real-time fMRI feedback engaged midbrain activation [76], whereas learned down regulation of the amygdala was associated with increased connectivity between amygdala and midbrain during the regulatory phase [75]. Furthermore, midbrain activation was also observed in patients with depression during up regulation of emotion networks, that included the insula and lateral prefrontal areas [74]. 

The here observed activations during emotion regulation are also in line with studies showing the activity of midbrain and dorsal AI activations associated with core affective states generation, thalamus, and ventral AI with the contextual representation of recalled emotion, whereas premotor regions and SMA with the maintenance of the emotional states [6]. Engen et al. (2017) also showed that a positive valence of endogenously-generated emotion was specifically associated with the VTA/SNG complex, whereas a negative valence with PAG. In this study, differential activations within upper mesencephalic regions for emotion polarities was not possible as participants reported to have recalled both positive and negative emotional memories. On the other hand, the PAG activity might more generally signal increased intensity of emotional memories. In fact, the PAG, although traditionally associated with pain perception, fear, and defensive behavior [7], seems also involved in positive emotional responses [77,78,79,80]. The activity of this region was observed during highly arousing states independently of emotion category [2] and it has been then proposed to mediate emotional arousal through its projections to other brainstem sites [7]. Conceivably, the PAG might support regulation of endogenously-induced emotions, and be relevant for both up and down emotion regulation. 

The upper midbrain activation does not appear to be directly associated with feedback assessment, as in this study, it was measured comparing up and down emotion regulation conditions, both presenting feedback information. Accordingly, a previous study aiming to investigate brain activity related to feedback control and monitoring during real-time fMRI-based emotion regulation did not report midbrain activity [81]. In short, current results suggest that the midbrain might mediate core affective states generation and regulation. 

Multivariate and univariate functional connectivity analyses also revealed extensive midbrain interactions with specific brain networks previously associated with self-induced emotion through recalling of emotionally salient autobiographical memories [6,82,83,84,85,86,87]. In particular, emotion regulation involved the SN, the default mode network (DMN), and the FPCN [88,89,90]. 

During increased emotional response, the upper midbrain positively correlated with the bilateral AI, with additional SN nodes of the mesencephalon, and with hippocampus, parahippocampal gyrus, thalamus, and basal ganglia; whereas a negative coupling was observed with ventromedial prefrontal regions. During the same condition, the right AI was positively coupled with several regions within the SN such as the left AI, amygdala, PAG, and SNG, and with thalamus; the anticorrelation was instead observed with the precuneus, medial, and dorsomedial prefrontal cortex. The reciprocal association between the midbrain and AI corroborates previous evidence of the involvement of these regions in modulating autonomic and emotional responses and their integration, possibly through descending projections from AI to the PAG [7,91]. In turn, mesothalamocortical projections to AI have been suggested to support essential aspects of emotion-related regulatory processes such as interoception and emotional awareness [16,92]. 

The analogous pattern of functional connectivity—that is a positive correlation of both midbrain and AI with other SN nodes and a negative correlation with frontal and parietal regions—was observed during both up and down regulation of emotion. However, the anticorrelation of both AI and midbrain with nodes of FPCN during up regulation resulted from reduced activity of the orbitofrontal cortex and medial prefrontal cortex concurrent to SN increased activation, whereas during decreased emotional response reduced SN activation was concurrent to increased FPCN involvement, as evidenced by the whole brain group analysis. This latter result is in line with previous studies showing top-down mechanisms actively contributing to the mitigation of affective states [22,23,93]. In fact, cognitive emotion regulation—both up and down regulation—is usually associated with increased activity of the prefrontal cortex [19,94]. On the contrary, the anticorrelation between SN and FPCN during up emotion regulation suggests that different regulatory mechanisms with respect to typical emotion regulation tasks might support this process. In this case, up emotion regulation appears to be mainly operated at a lower level through tight interactions between the midbrain and AI, possibly mediating the assessment of interoceptive predictive signals [95,96]. 

Additionally, SN activation corresponded to DMN deactivation, whereas Engen et al. (2017), in line with studies on internally-focused attention [97], observed a correlating activity between DMN and SN during increased intensity of the affective state. Here, the anticorrelation between SN and DMN might be related to the higher cognitive effort [89,98] necessary for assessing real-time fMRI feedback information during both up and down emotion regulation. 

In both up and down emotion regulation feedback of the AI activity, conveying information related to the actual emotional intensity level, permitted tracking (in)correctness of adopted regulation strategies, and thus represented a prediction error signal. Neurofunctional accounts of the AI, besides indicating its relevance for the assessment and integration of high level interoceptive inferences and physiological homeostatic signals [96], proposed its specific role in error-based learning of emotional states [99]. In this context, ascending dopaminergic prediction error signals [100,101] would be highly relevant for emotion regulation [10]. The extensive dopaminergic innervations [102] and high density of D1 dopamine receptors of the agranular insular cortex [103] can support regulation of emotion through the analysis of primary and secondary mappings of interoceptive signals. 

Furthermore, during both up and down emotion regulation, the AI and midbrain were positively coupled with the thalamus and striatum, areas that mediate reward-related brain signals during reinforcement learning [81,104,105,106,107,108]. A previous meta-analysis of real-time fMRI-based investigations indicated the anterior insula, striatum, anterior cingulate cortex, and lateral prefrontal cortex as crucial areas for learned control [108]. Although their specific role in emotion regulation remained unclear, mainly because of the heterogeneity of the studies included, these regions partially overlap with those related to the cognitive control of emotion, indicating some common substrates for both cognitive and real-time fMRI-based approaches to emotion regulation.

Overall, the whole brain midbrain connectivity analysis showed differential patterns of interactions during up and down emotion regulation. In particular, during up regulation, the functional integration among SN nodes would possibly mediate the reactivation of interoceptive and peripheral body signals related to emotional response, whereas the dissociation between SN and FPCN/DMN might suggest a reduced top-down inhibitory mechanism facilitating a more intense emotional expression. On the other hand, the SN and FPCN/DMN anticorrelation during down regulation might permit appropriate control of the emotional response, supported by the integrated activity of SN nodes, through specific attentional- and monitoring-related processes. Ultimately, the feedback of AI, a hub mediating assessment and integration of top-down emotion- and learning-related predictive signals with bottom up visceral and somatosensory information, would play a major role in attaining successful regulation of emotion. 

## 5. Limitations and Conclusions

The functional connectivity methods here adopted do not permit either estimation of the direct influence of a specific region over another one, also known as effective connectivity, or their potential modifications over time. Thus, several aspects related to the identified functional networks remained unclear. For instance, the nature of reciprocal connectivity between SN and FPCN, whether differential directional interactions characterize up and down regulation, and also the underlying dynamics of a nonstationary process [59,109] such as emotion regulation. 

A further limitation consists in the lack of additional peripheral physiological data or subjective evaluation of emotional intensity that might have complemented and corroborated the results. On the other hand, the observed modulation of neurophysiological activity in the AI concurrent to specific emotion-related mental strategies might be considered a good indicator of effective emotion generation and regulation.

In light of the current standard in neuroimaging, the sample size of this study appears relatively small. The adopted multimodal approach to data analysis, combining univariate and multivariate methods, aimed at increasing at best the statistical power and reliability of the results. Underpowered studies are often characterized by reduced sensitivity—correct true positives detection—and can result in a biased effect size estimation, however cluster-level inference appears less affected by the sample size and permits higher replicability [110]. Multiple comparisons correction based on the TFCE statistic has been shown to approximately double the sensitivity, but at the cost of increased spatial bias [111]. This might be particularly critical for accurate localization of BOLD activity within small mesencephalic areas, such as the PAG and VTA/SNG, and their functional subdivisions. Future fMRI studies aiming at investigating the role of the midbrain in emotion regulation would then substantially benefit from increased sample size and higher spatial resolution.

Despite these limitations, the present findings suggest that functional interactions within dopaminergic mesocorticolimbic systems might importantly contribute to emotion regulatory processes in humans. Finally, these results corroborate and extend previous investigations on emotion regulation based on real-time fMRI by indicating the efficacy of this approach in supporting modulation of self-generated affective states guided by the level of activation of core neural substrates of emotion.

## Figures and Tables

**Table 1:** BOLD activations during all real-time fMRI-based emotion regulation runs. The statistical threshold was set at *p* < 0.001 cluster level corrected using probabilistic threshold-free cluster enhancement (pTFCE).

Up > Down				
K	T	x, y, z (mm)	Hemisphere	Area
200	5.88	−8, 10, 65	L	SMA
92	5.62	45, −66, 0	R	Middle temporal gyrus
81	5.28	−37, 0, 50	L	Premotor cortex
62	5.14	5, −26, −10	R	Midbrain
39	5.03	18, −10, 25	R	Caudate nucleus
77	4.98	42, 0, 50	R	Premotor cortex
114	4.87	−37, 20, 0	L	Anterior insula
107	4.70	42, 13, 5	R	Anterior insula
47	4.19	2, −19, 5	R	Thalamus

## References

[1] Sheppes G., Suri G., Gross J.J. (2015). Emotion regulation and psychopathology. Annu. Rev. Clin. Psychol..

[2] Lindquist K.A., Wager T.D., Kober H., Bliss-Moreau E., Barrett L.F. (2012). The brain basis of emotion: A meta-analytic review. Behav. Brain Sci..

[3] Kragel P.A., LaBar K.S. (2016). Decoding the Nature of Emotion in the Brain. Trends Cogn. Sci..

[4] Parvizi J., Damasio A. (2001). Consciousness and the brainstem. Cognition.

[5] Buhle J.T., Kober H., Ochsner K.N., Mende-Siedlecki P., Weber J., Hughes B.L., Kross E., Atlas L.Y., McRae K., Wager T.D. (2013). Common representation of pain and negative emotion in the midbrain periaqueductal gray. Soc. Cogn. Affect. Neurosci..

[6] Engen H.G., Kanske P., Singer T. (2017). The neural component-process architecture of endogenously generated emotion. Soc. Cogn. Affect. Neurosci..

[7] Motta S.C., Carobrez A.P., Canteras N.S. (2017). The periaqueductal gray and primal emotional processing critical to influence complex defensive responses, fear learning and reward seeking. Neurosci. Biobehav. Rev..

[8] Venkatraman A., Edlow B.L., Immordino-Yang M.H. (2017). The Brainstem in Emotion: A Review. Front Neuroanat..

[9] Panksepp J. (2010). Affective neuroscience of the emotional BrainMind: Evolutionary perspectives and implications for understanding depression. Dialogues Clin. Neurosci..

[10] Salgado-Pineda P., Delaveau P., Blin O., Nieoullon A. (2005). Dopaminergic contribution to the regulation of emotional perception. Clin. Neuropharmacol..

[11] Badgaiyan R.D., Fischman A.J., Alpert N.M. (2009). Dopamine release during human emotional processing. Neuroimage.

[12] Etkin A., Egner T., Kalisch R. (2011). Emotional processing in anterior cingulate and medial prefrontal cortex. Trends Cogn. Sci..

[13] Linnman C., Moulton E.A., Barmettler G., Becerra L., Borsook D. (2012). Neuroimaging of the periaqueductal gray: State of the field. Neuroimage.

[14] Tucker D.M.D., Luu P.D., Joan C.B. (2000). Anatomy and physiology of human emotion: Vertical integration of brainstem, limbic and cortical systems. Series in Affective Science. The Neuropsychology of Emotion.

[15] Damasio A.R. (1996). The somatic marker hypothesis and the possible functions of the prefrontal cortex. Philos. Trans. R Soc. Lond. B Biol. Sci..

[16] Craig A.D. (2003). Interoception: The sense of the physiological condition of the body. Curr. Opin. Neurobiol..

[17] Seeley W.W., Menon V., Schatzberg A.F., Keller J., Glover G.H., Kenna H., Reiss A.L., Greicius M.D. (2007). Dissociable intrinsic connectivity networks for salience processing and executive control. J. Neurosci..

[18] Ochsner K.N., Bunge S.A., Gross J.J., Gabrieli J.D. (2002). Rethinking feelings: An FMRI study of the cognitive regulation of emotion. J. Cogn. Neurosci..

[19] Ochsner K.N., Ray R.D., Cooper J.C., Robertson E.R., Chopra S., Gabrieli J.D., Gross J.J. (2004). For better or for worse: Neural systems supporting the cognitive down- and up-regulation of negative emotion. Neuroimage.

[20] Ochsner K.N., Silvers J.A., Buhle J.T. (2012). Functional imaging studies of emotion regulation: A synthetic review and evolving model of the cognitive control of emotion. Ann. N. Y. Acad. Sci..

[21] Buhle J.T., Silvers J.A., Wager T.D., Lopez R., Onyemekwu C., Kober H., Weber J., Ochsner K.N. (2014). Cognitive reappraisal of emotion: A meta-analysis of human neuroimaging studies. Cereb. Cortex.

[22] Kohn N., Eickhoff S.B., Scheller M., Laird A.R., Fox P.T., Habel U. (2014). Neural network of cognitive emotion regulation--an ALE meta-analysis and MACM analysis. Neuroimage.

[23] Etkin A., Buchel C., Gross J.J. (2015). The neural bases of emotion regulation. Nat. Rev. Neurosci..

[24] Seymour B., O’Doherty J.P., Dayan P., Koltzenburg M., Jones A.K., Dolan R.J., Friston K.J., Frackowiak R.S. (2004). Temporal difference models describe higher-order learning in humans. Nature.

[25] Schiller D., Levy I., Niv Y., LeDoux J.E., Phelps E.A. (2008). From fear to safety and back: Reversal of fear in the human brain. J. Neurosci..

[26] Linhartova P., Latalova A., Kosa B., Kasparek T., Schmahl C., Paret C. (2019). fMRI neurofeedback in emotion regulation: A literature review. Neuroimage.

[27] Thibault R.T., MacPherson A., Lifshitz M., Roth R.R., Raz A. (2018). Neurofeedback with fMRI: A critical systematic review. Neuroimage.

[28] Caria A., Veit R., Sitaram R., Lotze M., Weiskopf N., Grodd W., Birbaumer N. (2007). Regulation of anterior insular cortex activity using real-time fMRI. Neuroimage.

[29] Ruiz S., Lee S., Soekadar S.R., Caria A., Veit R., Kircher T., Birbaumer N., Sitaram R. (2013). Acquired self-control of insula cortex modulates emotion recognition and brain network connectivity in schizophrenia. Hum. Brain Mapp..

[30] Linden D.E., Habes I., Johnston S.J., Linden S., Tatineni R., Subramanian L., Sorger B., Healy D., Goebel R. (2012). Real-time self-regulation of emotion networks in patients with depression. PLoS ONE.

[31] Cohen Kadosh K., Luo Q., de Burca C., Sokunbi M.O., Feng J., Linden D.E.J., Lau J.Y.F. (2016). Using real-time fMRI to influence effective connectivity in the developing emotion regulation network. Neuroimage.

[32] Paret C., Ruf M., Gerchen M.F., Kluetsch R., Demirakca T., Jungkunz M., Bertsch K., Schmahl C., Ende G. (2016). fMRI neurofeedback of amygdala response to aversive stimuli enhances prefrontal-limbic brain connectivity. Neuroimage.

[33] Grone M., Dyck M., Koush Y., Bergert S., Mathiak K.A., Alawi E.M., Elliott M., Mathiak K. (2015). Upregulation of the rostral anterior cingulate cortex can alter the perception of emotions: fMRI-based neurofeedback at 3 and 7 T. Brain Topogr..

[34] Veit R., Singh V., Sitaram R., Caria A., Rauss K., Birbaumer N. (2012). Using real-time fMRI to learn voluntary regulation of the anterior insula in the presence of threat-related stimuli. Soc. Cogn. Affect. Neurosci..

[35] Lee S., Ruiz S., Caria A., Veit R., Birbaumer N., Sitaram R. (2011). Detection of cerebral reorganization induced by real-time fMRI feedback training of insula activation: A multivariate investigation. Neurorehabil. Neural Repair.

[36] Moll J., Weingartner J.H., Bado P., Basilio R., Sato J.R., Melo B.R., Bramati I.E., de Oliveira-Souza R., Zahn R. (2014). Voluntary enhancement of neural signatures of affiliative emotion using FMRI neurofeedback. PLoS ONE.

[37] Sarkheil P., Zilverstand A., Kilian-Hutten N., Schneider F., Goebel R., Mathiak K. (2015). fMRI feedback enhances emotion regulation as evidenced by a reduced amygdala response. Behav. Brain Res..

[38] Marxen M., Jacob M.J., Muller D.K., Posse S., Ackley E., Hellrung L., Riedel P., Bender S., Epple R., Smolka M.N. (2016). Amygdala Regulation Following fMRI-Neurofeedback without Instructed Strategies. Front Hum. Neurosci..

[39] Koush Y., Meskaldji D.E., Pichon S., Rey G., Rieger S.W., Linden D.E., Van De Ville D., Vuilleumier P., Scharnowski F. (2017). Learning Control Over Emotion Networks Through Connectivity-Based Neurofeedback. Cereb. Cortex.

[40] Shibata K., Watanabe T., Kawato M., Sasaki Y. (2016). Differential Activation Patterns in the Same Brain Region Led to Opposite Emotional States. PLoS Biol..

[41] Caria A., Sitaram R., Veit R., Begliomini C., Birbaumer N. (2010). Volitional control of anterior insula activity modulates the response to aversive stimuli. A real-time functional magnetic resonance imaging study. Biol. Psychiatry.

[42] Sulzer J., Sitaram R., Blefari M.L., Kollias S., Birbaumer N., Stephan K.E., Luft A., Gassert R. (2013). Neurofeedback-mediated self-regulation of the dopaminergic midbrain. Neuroimage.

[43] MacInnes J.J., Dickerson K.C., Chen N.K., Adcock R.A. (2016). Cognitive Neurostimulation: Learning to Volitionally Sustain Ventral Tegmental Area Activation. Neuron.

[44] Greer S.M., Trujillo A.J., Glover G.H., Knutson B. (2014). Control of nucleus accumbens activity with neurofeedback. Neuroimage.

[45] Holland A.C., Kensinger E.A. (2010). Emotion and autobiographical memory. Phys. Life Rev..

[46] Touroutoglou A., Hollenbeck M., Dickerson B.C., Feldman Barrett L. (2012). Dissociable large-scale networks anchored in the right anterior insula subserve affective experience and attention. Neuroimage.

[47] Menon V. (2015). Salience Network. Brain Mapping: An Encyclopedic Reference.

[48] Caballero-Gaudes C., Reynolds R.C. (2017). Methods for cleaning the BOLD fMRI signal. Neuroimage.

[49] Togo H., Rokicki J., Yoshinaga K., Hisatsune T., Matsuda H., Haga N., Hanakawa T. (2017). Effects of Field-Map Distortion Correction on Resting State Functional Connectivity MRI. Front Neurosci..

[50] Worsley K.J., Friston K.J. (1995). Analysis of fMRI time-series revisited–Again. Neuroimage.

[51] Stelzer J., Lohmann G., Mueller K., Buschmann T., Turner R. (2014). Deficient approaches to human neuroimaging. Front Hum. Neurosci..

[52] Smith S.M., Nichols T.E. (2009). Threshold-free cluster enhancement: Addressing problems of smoothing, threshold dependence and localisation in cluster inference. Neuroimage.

[53] Spisak T., Spisak Z., Zunhammer M., Bingel U., Smith S., Nichols T., Kincses T. (2018). Probabilistic TFCE: A generalized combination of cluster size and voxel intensity to increase statistical power. Neuroimage.

[54] Poldrack R.A. (2007). Region of interest analysis for fMRI. Soc. Cogn. Affect. Neurosci..

[55] Cremers H.R., Wager T.D., Yarkoni T. (2017). The relation between statistical power and inference in fMRI. PLoS ONE.

[56] Brett M., Anton J.-L., Valabregue R., Poline J.-B. (2002). Region of Interest Analysis Using the MarsBar Toolbox for SPM 99. NeuroImage.

[57] MarsBar toolbox. http://marsbar.sourceforge.net.

[58] Gonzalez-Castillo J., Hoy C.W., Handwerker D.A., Robinson M.E., Buchanan L.C., Saad Z.S., Bandettini P.A. (2015). Tracking ongoing cognition in individuals using brief, whole-brain functional connectivity patterns. Proc. Natl. Acad. Sci. USA.

[59] Gonzalez-Castillo J., Bandettini P.A. (2018). Task-based dynamic functional connectivity: Recent findings and open questions. Neuroimage.

[60] Kaufmann T., Alnaes D., Brandt C.L., Doan N.T., Kauppi K., Bettella F., Lagerberg T.V., Berg A.O., Djurovic S., Agartz I. (2017). Task modulations and clinical manifestations in the brain functional connectome in 1615 fMRI datasets. Neuroimage.

[61] Laumann T.O., Snyder A.Z., Mitra A., Gordon E.M., Gratton C., Adeyemo B., Gilmore A.W., Nelson S.M., Berg J.J., Greene D.J. (2017). On the Stability of BOLD fMRI Correlations. Cereb. Cortex.

[62] Whitfield-Gabrieli S., Nieto-Castanon A. (2012). Conn: A functional connectivity toolbox for correlated and anticorrelated brain networks. Brain Connect..

[63] CONN functional connectivity toolbox. http://www.nitrc.org/projects/conn.

[64] Calhoun V.D., Adali T., Pearlson G.D., Pekar J.J. (2001). A method for making group inferences from functional MRI data using independent component analysis. Hum. Brain Mapp.

[65] BoxPLotR. http://shiny.chemgrid.org/boxplotr/.

[66] Rorden C., Brett M. (2000). Stereotaxic display of brain lesions. Behav. Neurol..

[67] Hofstetter C., Achaibou A., Vuilleumier P. (2012). Reactivation of visual cortex during memory retrieval: Content specificity and emotional modulation. Neuroimage.

[68] Cabeza R., Ciaramelli E., Olson I.R., Moscovitch M. (2008). The parietal cortex and episodic memory: An attentional account. Nat. Rev. Neurosci..

[69] Gandhi S.P. (2001). Memory retrieval: Reactivating sensory cortex. Curr. Biol..

[70] Andrews-Hanna J.R. (2012). The brain’s default network and its adaptive role in internal mentation. Neuroscientist.

[71] Leech R., Sharp D.J. (2014). The role of the posterior cingulate cortex in cognition and disease. Brain.

[72] Lou H.C., Luber B., Crupain M., Keenan J.P., Nowak M., Kjaer T.W., Sackeim H.A., Lisanby S.H. (2004). Parietal cortex and representation of the mental Self. Proc. Natl. Acad. Sci. USA.

[73] Yao S., Becker B., Geng Y., Zhao Z., Xu X., Zhao W., Ren P., Kendrick K.M. (2016). Voluntary control of anterior insula and its functional connections is feedback-independent and increases pain empathy. Neuroimage.

[74] Mehler D.M.A., Sokunbi M.O., Habes I., Barawi K., Subramanian L., Range M., Evans J., Hood K., Luhrs M., Keedwell P. (2018). Targeting the affective brain-a randomized controlled trial of real-time fMRI neurofeedback in patients with depression. Neuropsychopharmacology.

[75] Herwig U., Lutz J., Scherpiet S., Scheerer H., Kohlberg J., Opialla S., Preuss A., Steiger V.R., Sulzer J., Weidt S. (2019). Training emotion regulation through real-time fMRI neurofeedback of amygdala activity. Neuroimage.

[76] Kanel D., Al-Wasity S., Stefanov K., Pollick F.E. (2019). Empathy to emotional voices and the use of real-time fMRI to enhance activation of the anterior insula. Neuroimage.

[77] Panksepp J. (1998). Affective Neuroscience: The Foundations of Human and Animal Emotions.

[78] Blood A.J., Zatorre R.J. (2001). Intensely pleasurable responses to music correlate with activity in brain regions implicated in reward and emotion. Proc. Natl. Acad. Sci. USA.

[79] Noriuchi M., Kikuchi Y., Senoo A. (2008). The functional neuroanatomy of maternal love: mother’s response to infant’s attachment behaviors. Biol. Psychiatry.

[80] Georgiadis J.R., Reinders A.A., Paans A.M., Renken R., Kortekaas R. (2009). Men versus women on sexual brain function: Prominent differences during tactile genital stimulation, but not during orgasm. Hum. Brain Mapp.

[81] Paret C., Zahringer J., Ruf M., Gerchen M.F., Mall S., Hendler T., Schmahl C., Ende G. (2018). Monitoring and control of amygdala neurofeedback involves distributed information processing in the human brain. Hum. Brain Mapp.

[82] Pardo J.V., Pardo P.J., Raichle M.E. (1993). Neural correlates of self-induced dysphoria. Am. J. Psychiatry.

[83] Gemar M.C., Kapur S., Segal Z.V., Brown G.M., Houle S. (1996). Effects of self-generated sad mood on regional cerebral activity: A PET study in normal subjects. Depression.

[84] George M.S., Ketter T.A., Parekh P.I., Herscovitch P., Post R.M. (1996). Gender differences in regional cerebral blood flow during transient self-induced sadness or happiness. Biol. Psychiatry.

[85] Reiman E.M., Lane R.D., Ahern G.L., Schwartz G.E., Davidson R.J., Friston K.J., Yun L.S., Chen K. (1997). Neuroanatomical correlates of externally and internally generated human emotion. Am. J. Psychiatry.

[86] Kimbrell T.A., George M.S., Parekh P.I., Ketter T.A., Podell D.M., Danielson A.L., Repella J.D., Benson B.E., Willis M.W., Herscovitch P. (1999). Regional brain activity during transient self-induced anxiety and anger in healthy adults. Biol. Psychiatry.

[87] Damasio A.R., Grabowski T.J., Bechara A., Damasio H., Ponto L.L., Parvizi J., Hichwa R.D. (2000). Subcortical and cortical brain activity during the feeling of self-generated emotions. Nat. Neurosci..

[88] Laird A.R., Fox P.M., Eickhoff S.B., Turner J.A., Ray K.L., McKay D.R., Glahn D.C., Beckmann C.F., Smith S.M., Fox P.T. (2011). Behavioral interpretations of intrinsic connectivity networks. J. Cogn. Neurosci..

[89] Spreng R.N., Sepulcre J., Turner G.R., Stevens W.D., Schacter D.L. (2013). Intrinsic architecture underlying the relations among the default, dorsal attention, and frontoparietal control networks of the human brain. J. Cogn. Neurosci..

[90] Spreng R.N., Stevens W.D., Chamberlain J.P., Gilmore A.W., Schacter D.L. (2010). Default network activity, coupled with the frontoparietal control network, supports goal-directed cognition. Neuroimage.

[91] Behbehani M.M. (1995). Functional characteristics of the midbrain periaqueductal gray. Prog. Neurobiol..

[92] Gu X., Hof P.R., Friston K.J., Fan J. (2013). Anterior insular cortex and emotional awareness. J. Comp. Neurol..

[93] Ochsner K.N., Gross J.J., Gross J.J. (2014). The neural bases of emotion and emotion regulation: A valuation perspective. Handbook of Emotion Regulation.

[94] Holland A.C., Kensinger E.A. (2013). The neural correlates of cognitive reappraisal during emotional autobiographical memory recall. J. Cogn. Neurosci..

[95] Barrett L.F., Simmons W.K. (2015). Interoceptive predictions in the brain. Nat. Rev. Neurosci..

[96] Seth A.K. (2013). Interoceptive inference, emotion, and the embodied self. Trends Cogn. Sci..

[97] Spreng R.N., Mar R.A., Kim A.S. (2009). The common neural basis of autobiographical memory, prospection, navigation, theory of mind, and the default mode: A quantitative meta-analysis. J. Cogn. Neurosci..

[98] Buckner R.L., Krienen F.M., Yeo B.T. (2013). Opportunities and limitations of intrinsic functional connectivity MRI. Nat. Neurosci..

[99] Singer T., Critchley H.D., Preuschoff K. (2009). A common role of insula in feelings, empathy and uncertainty. Trends Cogn. Sci..

[100] Schultz W. (2007). Behavioral dopamine signals. Trends Neurosci..

[101] Glimcher P.W. (2011). Understanding dopamine and reinforcement learning: The dopamine reward prediction error hypothesis. Proc. Natl. Acad. Sci. USA.

[102] Gaspar P., Berger B., Febvret A., Vigny A., Henry J.P. (1989). Catecholamine innervation of the human cerebral cortex as revealed by comparative immunohistochemistry of tyrosine hydroxylase and dopamine-beta-hydroxylase. J. Comp. Neurol..

[103] Hurd Y.L., Suzuki M., Sedvall G.C. (2001). D1 and D2 dopamine receptor mRNA expression in whole hemisphere sections of the human brain. J. Chem. Neuroanat..

[104] Tobler P.N., Fiorillo C.D., Schultz W. (2005). Adaptive coding of reward value by dopamine neurons. Science.

[105] Bayer H.M., Glimcher P.W. (2005). Midbrain dopamine neurons encode a quantitative reward prediction error signal. Neuron.

[106] Friston K.J., Shiner T., FitzGerald T., Galea J.M., Adams R., Brown H., Dolan R.J., Moran R., Stephan K.E., Bestmann S. (2012). Dopamine, affordance and active inference. PLoS Comput. Biol..

[107] Dayan P., Niv Y. (2008). Reinforcement learning: The good, the bad and the ugly. Curr. Opin. Neurobiol..

[108] Emmert K., Kopel R., Sulzer J., Bruhl A.B., Berman B.D., Linden D.E.J., Horovitz S.G., Breimhorst M., Caria A., Frank S. (2016). Meta-analysis of real-time fMRI neurofeedback studies using individual participant data: How is brain regulation mediated?. Neuroimage.

[109] Zarghami T.S., Friston K.J. (2020). Dynamic effective connectivity. Neuroimage.

[110] Bossier H., Roels S.P., Seurinck R., Banaschewski T., Barker G.J., Bokde A.L.W., Quinlan E.B., Desrivieres S., Flor H., Grigis A. (2020). The empirical replicability of task-based fMRI as a function of sample size. Neuroimage.

[111] Noble S., Scheinost D., Constable R.T. (2020). Cluster failure or power failure? Evaluating sensitivity in cluster-level inference. Neuroimage.

